# Methods for fusing uncertain results obtained from different models in accident reconstruction

**DOI:** 10.1080/20961790.2019.1704474

**Published:** 2020-01-27

**Authors:** Tiefang Zou, Fenglin He

**Affiliations:** a School of Automobile and Mechanical Engineering, Changsha University of Science and Technology, Changsha, China; b Key Laboratory of Safety Design and Reliability Technology for Engineering Vehicle, Changsha University of Science and Technology, Changsha, China

**Keywords:** Forensic sciences, accident reconstruction, different uncertain results, Monte Carlo Method, Sub-Interval Technique, fusing

## Abstract

Considering that almost all existing solutions of fusing different reconstructed results require experts’ opinions and the issue of how to fuse probabilistic results and mixed results has not been discussed. Two solutions are proposed. The first is based on the Monte Carlo Method (FMCM), while the second is based on the Sub-Interval Technique (FSIT). The method based on FMCM generates sample points according to the distribution of each uncertain result firstly, and then gives out the cumulative distribution function of the final fused result by statistical analysis. The method based on FSIT gets the result fusion interval set according to lower and upper bounds of all interval results and a given length *d* of each sub-interval firstly, and then calculate the weighted matrix of the result fusion interval. As a result, the cumulative distribution function of the final fused result can also be given out by statistical analysis. Finally, three real accidents are given to demonstrate the methods of FMCM and FSIT, the results of which show that both work well in practice.

## Introduction

The occurrence of traffic accidents is affected by many aspects, such as road surface and pavement marking [1]. Traces left on the accident scene are the basis of accident reconstruction and hence the precision of traces has a great influence on the accident reconstruction result. Unfortunately, traces always cannot be measured precisely because they will vanish slowly under the influence of passers-by or other vehicles or the weather. In order to make the reconstructed result more precise and reliable, lots of reconstruction methods are proposed, such as methods based on the braking distance of the vehicle [2,3], the throw distance of the pedestrian [3–5], the deformation of the vehicle [6,7], the injury of the human body [8–10], simulations (almost all traces), videos and data from some attached active safety equipment [8,11–18]. Accordingly, many uncertainty analysis methods are also proposed [2,19–26]. By using these proposed methods, the reconstructed results can be obtained from the uncertain information of traces. Based on these existing researches, it is easy for people to reconstruct an accident by selecting appropriate accident reconstruction methods, and naturally a lot of uncertain reconstructed results will be obtained. How to describe those different results obtained from different accident reconstruction methods becomes the next issue. According to some operators, Zou et al. [27] has proposed two solutions recently, the first is to fuse different results into one result, while the other is to rank different results according to their credibility. The experts’ opinions are necessary in these two solutions. It is not hard to obtain experts’ opinions in major cases but it is hard and unnecessary to obtain experts’ opinions in regular cases. In addition, there are at least two kinds of uncertain results in reconstructing an accident [26], which are interval and probabilistic results. It means that three (interval, probabilistic and mixed) situations can be resulted in fusing uncertain results, but only interval results have been fused in Zou’s research. All these show that new methods are deserved to be studied.

## Problems description

In a traffic accident, the reconstructed result can be calculated by

(1) $$y_{i}=f_{i}(x),i=1,2,\ldots ,s$$

where *x* are traces, *f_i_* is an accident reconstruction method, *s* is the number of accident reconstruction methods and *y_i_* is the reconstructed result. Normally, *y_i_* represents the reconstructed velocity. There will be three situations in the fusing process.

Situation 1, only interval results need to be fused.

(2) $$G=g_{1}(y_{i}),i=1,2,\ldots ,s$$

where *y_i_* is the interval result, *g* is a fusing method.

Situation 2, only probabilistic results need to be fused.

(3) $$G=g_{2}(y_{j}),j=1,2,\ldots ,s$$

where *y_j_* is the probabilistic result.

Situation 3, the interval and probabilistic results need to be fused together.

(4) $$\begin{array}{c} G=g_{3}(y_{i},y_{j}),i=1,2,\ldots ,s_{1};j=1,2,\ldots ,s_{2}\\ s_{1}+s_{2}=s \end{array}$$

where *y_i_* is the interval result and *y_j_* is the probabilistic result.

Methods proposed in Zou’s research [27] can be employed to tackle with Situation 1 if experts’ opinions can be obtained. As for the other two situations, new methods need to be studied. Hence, in the present paper two fusing methods will be proposed based on the Monte Carlo Method and the Sub-Interval Technique.

## The fusing method based on the Monte Carlo Method

The Monte Carlo Method is widely used and successful in many fields [19,20]. It is used in the paper to fuse different uncertain results. For convenience, this method is referred to as FMCM for short. Three steps of the method are:

Step 1. To obtain the distribution of different uncertain results. In the case, the interval results are regarded as probabilistic results that are subject to uniform distribution.

Step 2. To generate sample points according to the distribution of each uncertain result. In order to make the final fused result stable, the sum of sample points of each uncertain result should be $\mathrm{EO}\times 10^{8}$, where *EO* is a weighting coefficient vector. In regular cases, *EO* = 1/*s*; while in those major cases with experts’ opinions, *EO* is calculated from experts’ opinions shown in reference [27].

Step 3. To do statistical analysis. Firstly, all sample points generated in Step 2 are put together to form a new sample set. Obviously, there will be 10^8^ sample points in the new set. Then the cumulative distribution function of the final fused result will be given out by statistical analysis.

## The fusing method based on the Sub-Interval Technique

In reference [28], the FMCM is precise but not most efficient. The relatively more efficient Sub-Interval Technique [28] can replace it in many cases, with result quite similar to that from FMCM, if there are enough subintervals in the analysis. Hence another method based on Sub-Interval Technique referred to as FSIT for short, will be proposed. Four steps of the method are:

Step 1. To find out the interval of each uncertain result. As for a probabilistic result, its interval is formed by the lower and upper bound.

Step 2. To obtain the result fusion interval set. Firstly, a new interval [*a*, *b*] should be obtained based on the lower bound *a* and upper bound *b* obtained in Step 1. After that, the length *d* of those sub-intervals in the interval set can be given according to the maximum number of decimal places of those interval bounds in Step 1. As for a bound with *n* decimal places, the *d* should be smaller than 10^-*n*^. In fact, *d* is often set to 0.01. For example, as for two interval results {[1,3],[2,4]}, their result fusion interval sets can be given as {[1,1.01],…,[3.99,4]} when *d* = 0.01.

Step 3. To calculate the weighted matrix. The weighted matrix is show in Table 1.

**Table 1.** The weighted matrix.

	Sub-intervals		
Results	[*a, a* + *d*]*	…	[*b–d, b*]
1	*w*_11_	…	*w*_1_ *_k_*
…	…	…	…
*s*	*w*_*s*1_	…	*w_sk_*
Sum	*s*_1_	…	*s_k_*

* Where *w_ij_* is the weighted coefficient, and *k* = (*b*–*a*)/*d*, *s_i_*=*w*_1*i*_ +…+ *w_si_*.

As for an interval result [*R_i-lower_*, *R_i-upper_*] and a sub-interval [*c_j_*, *c_j_+d*], the weighted coefficient of each sub-interval is calculated by

(5) $$\begin{array}{c} w_{ij}=EO\times \frac{d}{R_{i-\mathrm{upper}}-R_{i-\text{lower}}},\text{if}\;c_{j}>R_{i-\mathrm{lower}}\;\\ \text{and}\;c_{j}+d\leq R_{i-\mathrm{upper}},\mathrm{else}\;w_{ij}=0 \end{array}$$

Where *EO* is the defined vector in the Step 2 of FMCM.

As for a probabilistic result *x* with an interval [*R_i-lower_*, *R_i-upper_*], a probabilistic density function *PDF*, and a sub-interval [*c_j_*, *c_j_+d*], the weighted coefficient of each sub-interval is calculated by

(6) $$\begin{array}{c} w_{ij}=EO\times \int_{c_{j}}^{c_{j}+d}\mathrm{PDF}(x)dx,\text{if}\;c_{j}>R_{i-\mathrm{lower}}\;\\ \text{and}\;c_{j}+d\leq R_{i-\mathrm{upper}},\mathrm{else}\;w_{ij}=0 \end{array}$$

Step 4. To do statistical analysis. The cumulative distribution function of the final fused results will be given out by statistical analysis.

## Case 1

The aim of this case is to show how to fuse interval results by the two proposed methods and validate the results by comparing them with the existing fusing results. There are four interval results and the weight of each result has been calculated through seven experts’ opinions [27]. Hence the *EO* can be given in Table 2 accordingly. FMCM and FSIT will then be used to fuse the interval results.

**Table 2.** The *EO* and interval results in Case 1.

Interval results	*EO*
[64, 66]	0.2970
[63, 65]	0.2769
[59, 69]	0.2315
	[65, 75]	0.1945

### To fuse interval results by FMCM

Step 1. The interval results are shown in Table 2, which are regarded as the probabilistic results subject to uniform distribution.

Step 2. To make the sum of sample points of each uncertain result equals $\mathrm{EO}\times 10^{8}$, and *EO* is shown in Table 2. Then sample points can be generated according to the distribution of each uncertain results.

**Figure 1.**
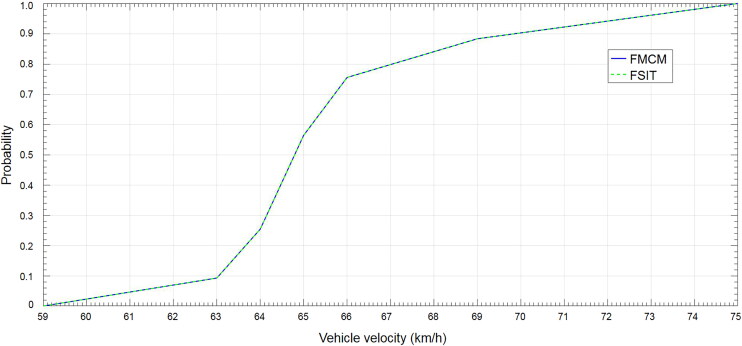
Cumulative distribution in Case 1. FMCM: Monte Carlo Method; FSIT: Sub-Interval Technique.

Step 3. To do statistical analysis. Firstly, all sample points generated in Step 2 are put together to form a new sample set. Obviously, there will be 10^8^ sample points in the new set. Then the cumulative distribution function of the final fused result will be given out by statistical analysis (Figure 1). It takes 1.95 s to complete the calculation.

### To fuse interval results by FSIT

Step 1. The interval results are also shown in the Table 2.

Step 2. Firstly, a new interval [59, 75] is obtained based on the lower bound and upper bound obtained in Step 1. Then the result fusion interval set {[59, 59.01],…,[74.99, 75]} with *d =* 0.01 is obtained.

Step 3. To calculate the weighted matrix by Eq. (5), and *EO* is shown in Table 2. Results are shown in Table 3.

**Table 3.** The weighted matrix in Case 1.

	Sub-intervals								
Results	[59, 59.01]	...	[63, 63.01]	...	[64, 64.01]	...	[65, 65.01]	...	[74.99, 75]
[64, 66]	0	...	0	...	0.000149	...	0.000149	...	0
[63, 65]	0	...	0.001385	...	0.001385	...	0	...	0
[59, 69]	0.000232	...	0.000232	...	0.000232	...	0.000232	...	0
[65, 75]	0	...	0	...	0	...	0.000195	...	0.000195
Sum	0.000232	...	0.001616	...	0.001765	...	0.000575	...	0.000195

Step 4. To do statistical analysis. The cumulative distribution of the fused result is given by doing statistical analysis (Figure 1). It takes 0.06 s to complete the calculation.

From Figure 1, the probability of any velocity interval can be seen clearly. For example, the probability of 59 < *v* < 70 km/h is about 90%. Besides, it is obvious that results obtained from the two methods are the same. However, the simulation time of FSIT is far less than that of FMCM.

### Comparison

**Figure 2.**
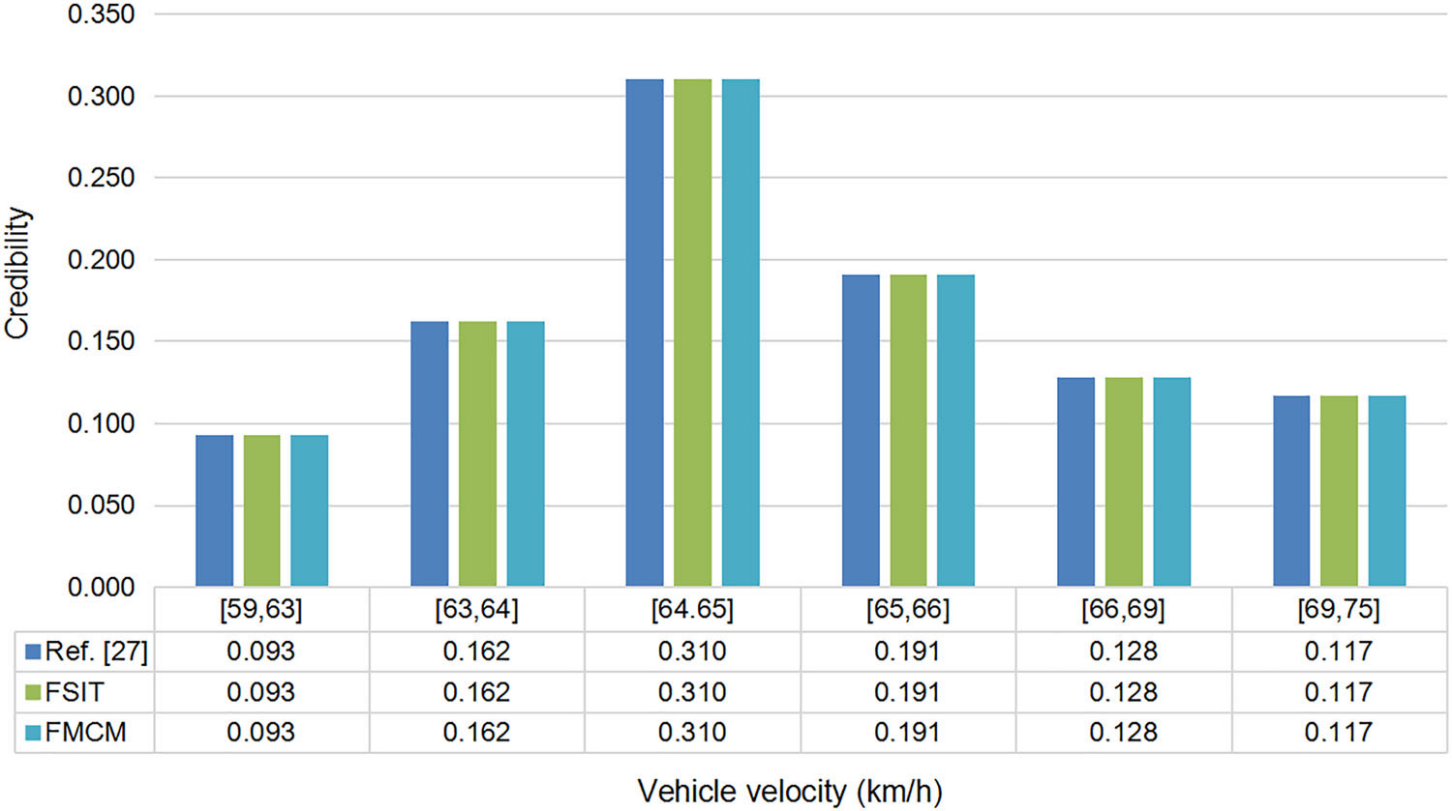
The fusion results of different methods. FMCM: Monte Carlo Method; FSIT: Sub-Interval Technique.

A specific fusion result interval set is given in reference [27], which is {[59,63],[63,64],[64,65],[65,66],[66,69],[69,75]}. In order to compare fusion results obtained by FMCM and FSIT with reference [27], the probability in the specific fusion result interval set is calculated again by the two proposed methods, and results are shown in Figure 2, from which we can intuitively draw a conclusion that results obtained by FMCM and FSIT are completely consistent with the fusion result in reference [27], which fully demonstrates that FMCM and FSIT are feasible and trustworthy.

## Case 2

The aim of this case is to show how to fuse probabilistic results. In reconstructing a car–pedestrian accident, two probabilistic results subject to normal distribution are obtained, and results are shown in Table 4. Detailed information about the case and the process of getting these results can be found in Supplement 1. FMCM and FSIT will then be used to fuse the two different results.

**Table 4.** Two probabilistic results.

Model	Mean	Variance	Interval
Model A	33.10	8.052	[17, 47]
Model B	14.02	4.202	[8, 21]

Model A: reconstruct the velocity based on the post-braking-distance.

Model B: reconstruct the velocity based on the throw distance of pedestrian.

### To fuse probabilistic results by FMCM

Step 1. The probabilistic results are shown in Table 4, which are subject to normal distribution.

Step 2. To make *EO* = 0.5 and the sum of sample points of each uncertain result equals $\mathrm{EO}\times 10^{8}$, and then sample points are generated according to its corresponding distribution.

**Figure 3.**
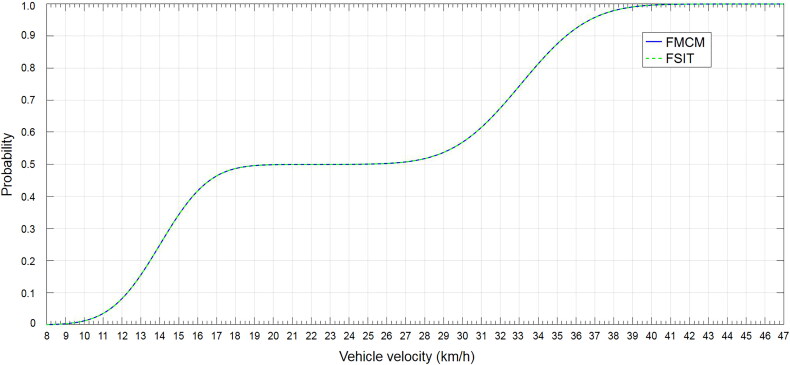
Cumulative distribution in Case 2. FMCM: Monte Carlo Method; FSIT: Sub-Interval Technique.

Step 3. To put all sample points generated in Step 2 together to form a new sample set and then give out the cumulative distribution function of the final fused result by statistical analysis (Figure 3). It takes 16.65 s to complete the calculation.

### To fuse probabilistic results by FSIT

Step 1. The interval of each uncertain result is shown in Table 4.

Step 2. A new interval [8, 47] is obtained based on the lower bound and upper bound obtained in Step 1. Then the result fusion interval set can be given as {[8,8.01],…,[46.99,47]} with *d =* 0.01.

Step 3. To calculate the weighted matrix by Eq. (6) with *EO* = 0.5. The weighted matrix is shown in Table 5.

**Table 5.** The weighted matrix in Case 2.

	Sub-interval						
Results	[8, 8.01]	...	[17, 17.01]	...	[21, 21.01]	...	[46.99, 47]
[17, 47]	0	...	0	...	8.2E-08	...	4.23E-09
[8, 21]	1.346E-05	...	3.328E-04	...	0	...	0
Sum	1.346E-05	...	3.328E-04	...	8.2E-08	...	4.23E-09

Step 4. To do statistical analysis. The cumulative distribution of the fused result can be given by doing statistical analysis (Figure 3). It takes 0.11 s to complete the calculation.

From Figure 3, the probability of any vehicle velocity interval can be seen clearly. For instance, the probability of 8<*v* < 25 km/h is about 50%. It can be easily concluded that results obtained from the two different methods are the same, but the simulation time of the FSIT is far less than the time of FMCM.

## Case 3

The aim of this case is to show how to fuse mixed uncertain results. Three interval results and a probabilistic result are obtained in reconstructing a car– pedestrian accident (Table 6). Detailed information of the case and the process of obtaining these results can be found in Supplement 2. Next, FMCM and FSIT are used to fuse these uncertain results.

**Table 6.** The uncertain mixed results.

Model	Results		
1	[45, 55]		
2	[55, 58]		
3		[45, 51]	
4*			[44, 59]

* The probability result obtained from Model 4, Mean = 52.06 km/h, Variance = 2.0725, Interval = [44, 59] km/h.

### To fuse mixed results by FMCM

Step 1. All results are shown in Table 6, where interval results are regarded as results subject to uniform distribution and the probabilistic result subject to normal distribution.

Step 2. The sum of sample points of each uncertain result is set to $\mathrm{EO}\times 10^{8}$, and *EO* = 0.25. Then sample points are generated according to its corresponding distribution.

**Figure 4.**
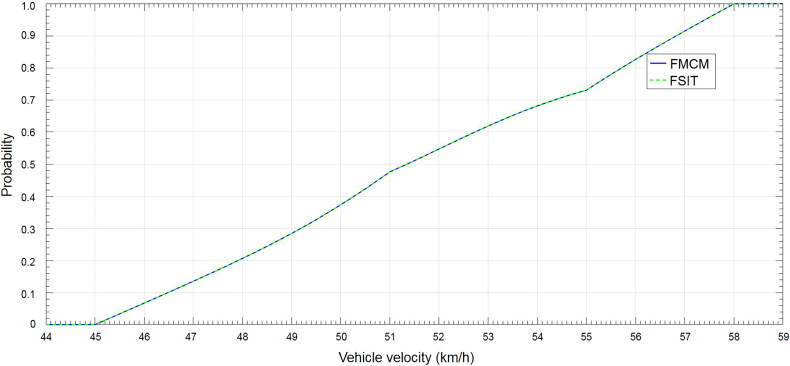
Cumulative distribution in Case 3. FMCM: Monte Carlo Method; FSIT: Sub-Interval Technique.

Step 3. To put all sample points generated in Step 2 together to form a new sample set and then give out the cumulative distribution function of the fused result by statistical analysis (Figure 4). It takes 105.7 s to complete the calculation.

### To fuse mixed results by FSIT

Step 1. The interval of each uncertain result is shown in Table 6.

Step 2. A new interval [44, 59] is obtained based on the lower bound and upper bound obtained in Step 1. Then the length *d* is set to 0.01, and *EO* = 0.25. The result fusion interval set is obtained (Table 7).

**Table 7.** The weighted matrix in Case 3.

	Sub-interval								
Results	[44, 44.01]	...	[45, 45.01]	...	[54, 54.01]	...	[55, 55.01]	...	[58.99, 59]
[45, 55]	0	...	0.0002	...	0.0002	...	0	...	0
[55, 58]	0	...	0	...	0	...	0.0006667	...	0
[45, 51]	0	...	0.0003333	...	0	...	0	...	0
[44, 59]	0.00000022593	...	0.0000012936	...	0.0002411	...	0.0001346	...	0.0000012936
Sum	0.00000022593	...	0.000534594	...	0.0004411	...	0.0008013	...	0.0000012936

Step 3. As for interval results, the weighted matrix is calculated by Eq. (5). As for the probabilistic result, the weighted matrix is calculated by Eq. (6). The final obtained weighted matrix is shown in Table 7.

Step 4. To give out the cumulative distribution function of the final fused results by doing statistical analysis (Figure 4). It takes 0.094 s to complete the calculation.

From Figure 4, the probability of any velocity interval can be seen clearly. For example, the probability of 48<*v* < 59 km/h is about 80%. It can be easily conclude, that results obtained from the two different methods are the same, but the simulation time of the FSIT is far less than the time of FMCM.

## Conclusions

From the analysis above, conclusions can be given below:

- The results obtained by FMCM and FSIT are completely consistent with the results shown in reference [27], which fully demonstrates that FMCM and FSIT are feasible and trustworthy.
- FMCM and FSIT can fuse interval, probabilistic and mixed uncertain results and they can work well no matter whether experts’ opinions are obtained or not.
- Research results show that both FMCM and FSIT can obtain results in a short time. The simulation time of FMCM typically varies from a few seconds to a few minutes, while that of FSIT is less than a second, which indicates that FSIT is more efficient.
- Both FMCM and FSIT can give out the cumulative distribution function of the final fused results by doing statistical analysis. The probability of any speed interval can be obtained from the cumulative distribution. This is meaningful and valuable in practice.

## Authors’ contributions

Tiefang Zou proposed the idea of the paper, wrote the paper and carried out simulations and calculations. Fenglin He was in charge of all calculations in the whole paper.

## Supplementary Material

Supplemental Material
