# Supplementary material for: Methods for fusing uncertain results obtained from different models in accident reconstruction
Source: Forensic Sci Res. 2020 Jan 27;7(2):272–8. doi: 10.1080/20961790.2019.1704474 (PMC9245986; doi:10.1080/20961790.2019.1704474)
Supplement: Supplemental Material [file TFSR_A_1704474_SM2791.docx]

# Supplement 1: Case 2

In the afternoon of 1st, November, 2015, a man whose height is 165cm and weight is 65kg was knocked down by a Mondeo on a dry side street in China. The sketch of the accident scene is shown in Figure. A1. According to the investigation of police officers, the center of gravity (CG) height of the pedestrian is about 0.9 m, the interval is [0.8,1] m, the throw distance of the pedestrian is about 3m, and the interval is [2,4] m. In addition, the mass of Mondeo is 1 561 kg, the post-braking-distance is about 5 m, and the interval is [4,6] m. The next assignment is to reconstruct the impact velocity.


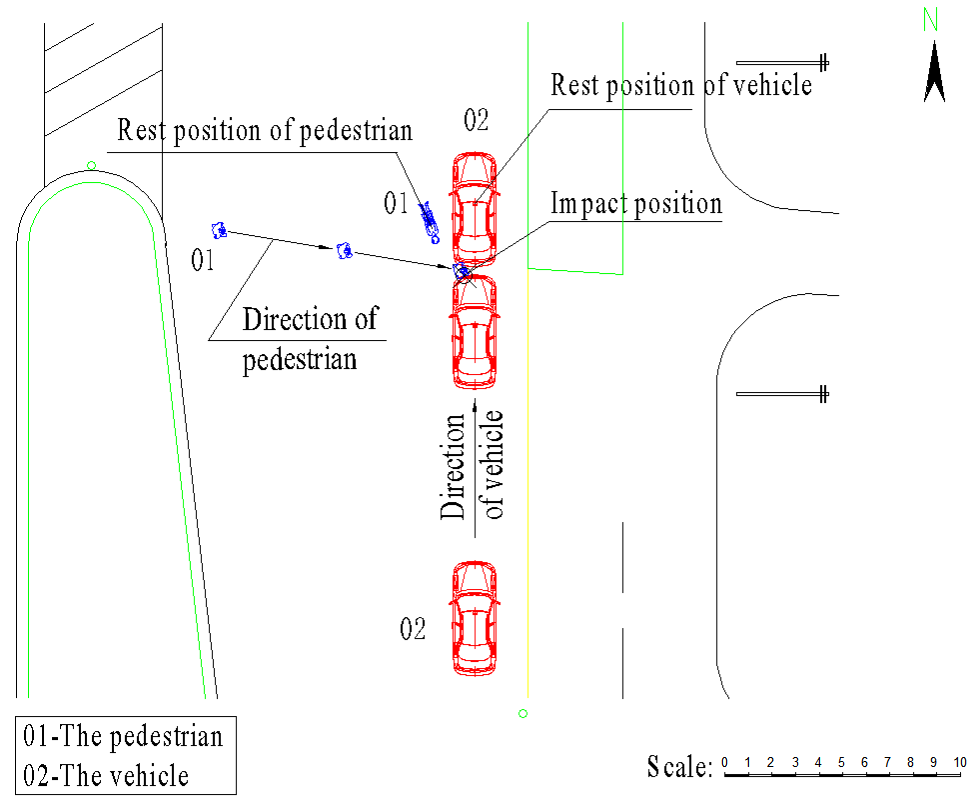


Figure A1. The sketch of the accident scene in Case 2.

# A1. Model A: Reconstruct the velocity based on the post-braking-distance

The empirical formula based on the post-braking-distance[3] is

$v=\frac{m_{v}+m_{p}}{m_{v}}\sqrt{2f_{v}gS_{v}}$ (A-1)

Where *v* is the impacted velocity, m/s; *m_v_* is the mass of vehicle, kg; *m_p_* is the weight of pedestrian, kg; *f_v_* is the vehicle-ground friction drag coefficient; *S_v_* is the post-braking-distance, m; g=9.8 m/s^2^.

In the accident, *m_v_=*1 561kg, *m_p_=*65kg, and *S_v_=*[4,6] m. According to existing researches[3, 26], the vehicle-ground friction drag coefficient is subject to normal distribution with the mean value 0.8 and the standard deviation 0.1, so the Monte Carlo Method can be applied to obtain the probabilistic result. The sum of sample points is set to 10^8^. Then the impacted velocity of the car can be calculated according to Eq. (A-1). The mean value is 33.08 km/h, the variance 8.052 and the interval is [17,47] km/h.

# A2. Model B: Reconstruct the velocity based on the throw distance of pedestrian

The empirical formula based on the throw distance of pedestrian[4, 5] is

$v=3.6\times\sqrt{2g}\times\mu\times\left(\sqrt{h+S_{p}/\mu}-\sqrt{h}\right)$ (A-2)

Where *v* is the impacted velocity, km/h; g=9.8 m/s^2^; *μ* is the coefficient between the pedestrian and the ground; *h* is the CG height of pedestrian; *S_p_* is the throw distance of the pedestrian.

In the accident, *h*=[0.8,1] m, and *S_p_*=[2,4] m. According to existing researches[3, 26], the coefficient between the pedestrian and the ground is subject to normal distribution with the mean value 0.6 and the standard deviation 0.1, so the Monte Carlo Method can be applied to obtain the probabilistic result. The sum of sample points is set to 10^8^. Then the impacted velocity of the car can be calculated according to Eq. (A-2), with means value 14.02 km/h, variance 4.202, and the interval [8,21] km/h.

# A3. Results of Case 2

All the results of Case 2 are shown in Table A1.

Table A1. Two probabilistic results (km/h).

| Model | Mean | Variance | Interval |
| --- | --- | --- | --- |
| Model A | 33.08 | 8.052 | [17,47] |
| Model B | 14.02 | 4.202 | [8,21] |

Model A: reconstruct the velocity based on the post-braking-distance; Model B: reconstruct the velocity based on the throw distance of pedestrian

# Supplement 2: Case 3

In the afternoon of March 6, 2015, a man whose height is 180 cm and weight is 74kg was knocked down by a Jetta on a dry side street in China. The sketch of the accident scene is shown in Figure B1. Based on the investigation of police officers, the throw distance of the pedestrian is about 24m, with the interval [23,25] m, the relative rest position of vehicle and pedestrian about 6m, and the interval [5,7] m. The CG height of the pedestrian is about 0.9m, and the interval is [0.8,1] m. The next assignment is to reconstruct the impact velocity.


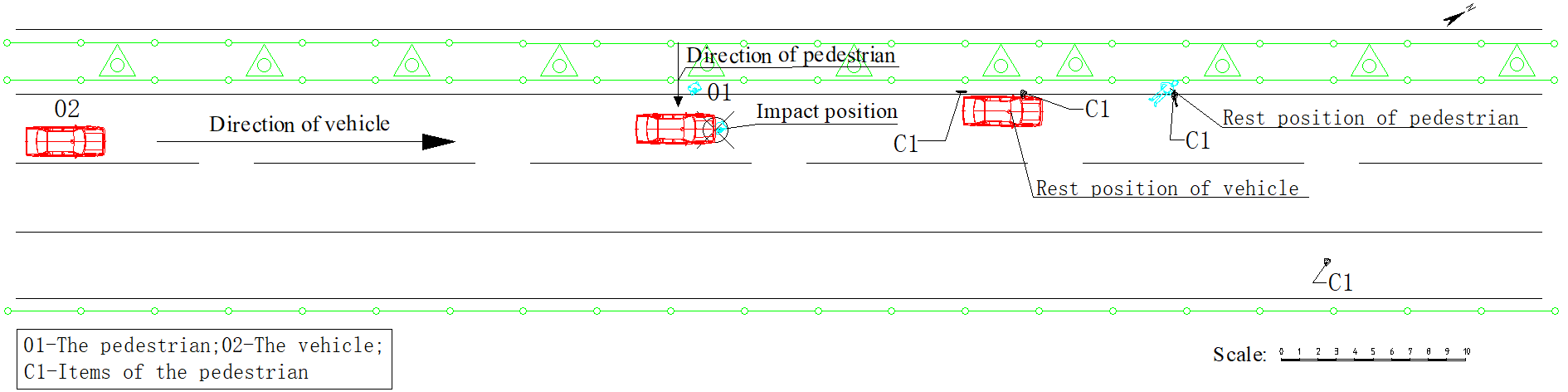


Figure B1. The sketch of the accident scene in Case 3.

# B1. Model A: Reconstruct the velocity based on PC-Crash

Firstly, the accident scene in simulation is built by putting the Figure B1 into PC-Crash. Then the default model of the pedestrian and the vehicle in PC-Crash is imported. After that, the pedestrian and the vehicle model in simulation are built by revising some parameters according to the truth value. Finally, the simulation result is obtained by simulating many times. Multiple attempts reveal that traces in simulation are mostly accord with traces left in the accident scene when the impacted velocity of vehicle is 50km/h and the speed of the pedestrian is 5km/h. The simulation result is shown in Figure B2. It can be easily found that both the rest positions of the vehicle and the pedestrian and the impact position in simulation are well accord with actual positions.

The injury information not used above can be used to validate simulation. The injury information of different body parts of the pedestrian in simulation is shown in Table B1. The HIC15 is 2600, greater than 700, which may be the most likely reason why the pedestrian is dead in this accident. The relative position of the vehicle and the pedestrian in simulation that are shown in Figure B3 when *t*=0.13 s and *t*=1.165 s can explain the death of the pedestrian well.

Now, we can conclude that the impact velocity in the accident is about 50km/h. Considering that traces in the accident are uncertain and the result in simulation is also uncertain (An obvious example is that the rest position of the pedestrian is not the same as that in the sketch map), Zou’s research[25] should be employed here to analyze the uncertainty of the simulation result. Finally, the interval result can be obtained, which is [45,55] km/h.


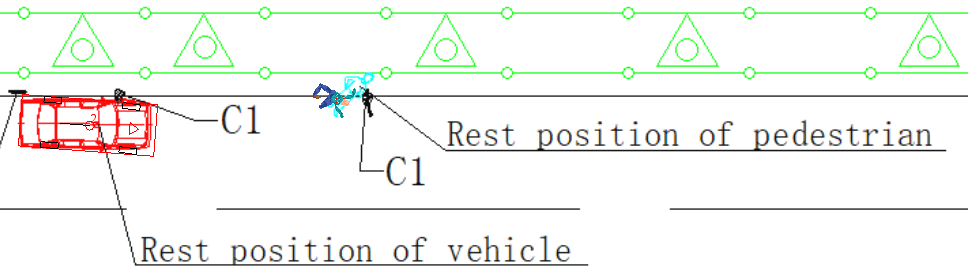


Figure B2. The simulation result in Case 3.

Table B1. The deductive injury of different body parts of the pedestrian.

| References | Parts of body | Injury criterial | Max simulation value | Deductive injury |
| --- | --- | --- | --- | --- |
| [28-30] | Head | HIC≤700 | HIC15=2600 | Fatal injury |
| [29, 30] | Torso | 3 ms≤60 *g* | 16.71 *g* | No fracture |
| [30, 31, 32] | Femur | Shearing force＜6.3k N | Femur left=2665.7 N  Femur right=2006.2 N | No fracture |
| [30-32] | Lower leg | Shearing force＜4k N | Left lower leg=953.9 N  Right lower leg=710.9 N | No fracture |


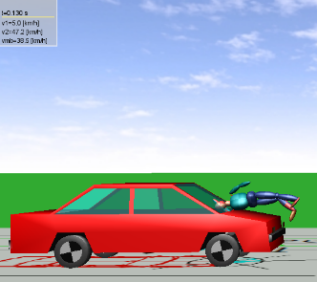

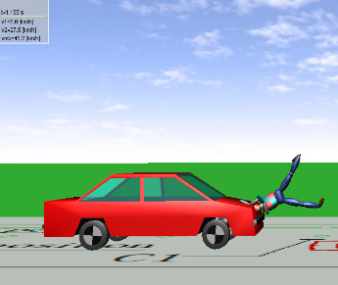


Figure B3. The relative position of the vehicle and the pedestrian in simulation.

# B2. Model B: Reconstruct the velocity based on Fugger Method

The Fugger model[33] is:

$v=8.3604S_{p}^{0.6046}$ (B-1)

Where *v* is the impact velocity; *S_p_* is the throw distance of the pedestrian.

In the accident, *S_p_*= [23,25] m, so the upper and lower bound method can be applied to obtain the interval result, which is [55,58] km/h.

# B3. Model C: A regression equation based on the relative rest position of vehicle and pedestrian

The regression equation based on the relative rest position of vehicle and pedestrian[34] is

$v=5.495D-90.676H+30.038L+30.412H_{p}+5.89$ (B-2)

Where *D* is the relative rest position of the vehicle and the pedestrian; *H* is the height of vehicle front; *L* is the length of vehicle front; *H_p_* is the height of the pedestrian.

According to the investigation of police, *D*=[5,7]m; the height of the vehicle front is 0.8m; the length of the vehicle front is 0.82m; and the height of the pedestrian is 1.8m. Hence, the upper and lower bound method can also be applied to obtain the interval result, which is [45,51] km/h.

# B4. Model D: Reconstruct the velocity based on the throw distance of the pedestrian

The empirical formula based on the throw distance of pedestrian[4, 5] is

$v=3.6\times\sqrt{2g}\times\mu\times\left(\sqrt{h+S_{p}/\mu}-\sqrt{h}\right)$ (B-3)

Where *v* is the impacted velocity, km/h; g=9.8 m/s^2^; *μ* is the coefficient between the pedestrian and the ground; *h* is the CG height of pedestrian; *S_p_* is the throw distance of the pedestrian.

In the accident, *h*=[0.8,1] m, and *S_p_*=[23,25] m. According to existing researches[3, 26], the coefficient between the pedestrian and the road is subject to normal distribution with the mean value 0.6 and the standard deviation 0.1, so the Monte Carlo Method can be applied to obtain the probabilistic result. The sum of sample points is set to 10^8^. Then the impacted velocity of the car can be calculated according to Eq. (B-3). The mean value is 52.06 km/h, the variance is 2.0725 and the interval is [44,59] km/h.

# B5. Results of Case 3

All the results of Case 3 are shown in Table B2.

Table B2. The uncertain mixed results (km/h).

| Model 1 | Model 2 | Model 3 | Model 4* |
| --- | --- | --- | --- |
| [45,55] | [55,58] | [45,51] | [44,59] |

*The probabilistic result obtained from Model 4, Mean=52.06 km/h, Variance=2.0725, Interval= [44,59] km/h
